# Enhancement of the Surface Morphology of (Bi_0.4_Sb_0.6_)_2_Te_3_ Thin Films by In Situ Thermal Annealing

**DOI:** 10.3390/nano13040763

**Published:** 2023-02-17

**Authors:** Liesbeth Mulder, Hanne van de Glind, Alexander Brinkman, Omar Concepción

**Affiliations:** 1 MESA+ Institute for Nanotechnology, University of Twente, 7500 AE Enschede, The Netherlands; 2 Peter Grünberg Institute (PGI-9), Forschungszentrum Juelich, 52425 Juelich, Germany

**Keywords:** topological insulator, smooth surfaces, molecular beam epitaxy, in situ thermal post anneal, (Bi_1−x_Sb_x_)_2_Te_3_

## Abstract

The study of the exotic properties of the surface states of topological insulators requires defect-free and smooth surfaces. This work aims to study the enhancement of the surface morphology of optimally doped, high-crystalline (Bi_0.4_Sb_0.6_)_2_Te_3_ films deposited by molecular beam epitaxy on Al_2_O_3_ (001) substrates. Atomic force microscopy shows that by employing an in situ thermal post anneal, the surface roughness is reduced significantly, and transmission electron microscopy reveals that structural defects are diminished substantially. Thence, these films provide a great platform for the research on the thickness-dependent properties of topological insulators.

## 1. Introduction

Three-dimensional (3D) topological insulators (TIs) are of great interest for future applications in the field of low-power electronics [1], spintronics, and quantum computing [2] due to their intrinsic electronic properties. These materials exhibit a bulk band gap and display topological conducting surface states, which are characterized by a unique coupling between the spin and the momentum of an electron. Quantum mechanically, these surface states are described by a wave function that decays into the bulk material on the length scale of just a few unit cells. The topological surface states of opposite sides can interact with each other as the material thickness is decreased. The overlapping surface states give rise to a hybridization gap [3,4]. Depending on the thickness of the 3D TI, the system is predicted to alternate between being a trivial insulator or having conducting edge states and showing the quantum spin Hall (QSH) effect. This crossover between different topological states is modeled to occur on the level of a few unit cells [5]. To obtain experimental insight into the influence of the TI thickness on these electronic properties, it is crucial to have defect-free and ultra-flat films with an optimized degree of uniformity.

(Bi_1−x_Sb_x_)_2_Te_3_ compounds exhibit topological surface states with a single Dirac cone centered around the high-symmetry Γ point in the Brillouin zone. The doping level, *x*, can be optimized to position both the Dirac point and Fermi energy in the bulk band gap. (Bi_1−x_Sb_x_)_2_Te_3_ has a rhombohedral crystal structure with a unit cell built out of quintuple layers (QLs). These QLs are approximately 1 nm high and coupled to each other by weak van der Waals (VdW) bonds in the [001] direction. The growth mechanism of (Bi_1−x_Sb_x_)_2_Te_3_ allows for characteristic triangular terraced islands that exhibit height differences of single QL steps. These well-defined height differences make (Bi_1−x_Sb_x_)_2_Te_3_ a perfect candidate for a height-dependent study of the influence of the hybridization gap on the TI’s electronic properties.

The growth of (Bi_1−x_Sb_x_)_2_Te_3_ has been reported using different techniques such as molecular beam epitaxy (MBE) [6], electron beam evaporation [7], sputtering [8], electrodeposition [9], pulsed laser deposition [10], and others. Among these deposition techniques, MBE stands out for the precise control it offers over various growth parameters, allowing to grow thin films with a specific stoichiometry and thickness. Additionally, given the VdW nature of the material, it is possible to use a wide variety of substrates for its growth, such as Al_2_O_3_ [11], GaAs [12], InP [13], Si [14], and SrTiO_3_ [15]. Al_2_O_3_ substrates were chosen in this study since they are highly insulating (which is ideal for transport measurements), are easy to handle and clean, and are readily commercially available.

This work aims to maximize the lateral terrace dimensions to allow the creation of devices that mainly consist of a uniform film thickness. For this study, we use the optimally doped TI (Bi_0.4_Sb_0.6_)_2_Te_3_ (BST) deposited on Al_2_O_3_ (001) substrates using MBE. By selecting substrates with a proper miscut, we are able to suppress one of the twin domains in the BST films without applying any additional improvement procedure. By employing an in situ thermal anneal we acquire smooth, ultra-thin films that create a perfect platform to detect signatures of the hybridization gap.

## 2. Materials and Methods

The BST films studied were deposited by MBE on Al_2_O_3_ (001) substrates in an ultra-high vacuum Octoplus 300 MBE System from Dr. Eberl MBE Komponenten, with a base pressure of about 5.0 × 10^−11^ mbar. The standard growth temperature used during deposition was 225 °C. Using standard Knudsen effusion cells, high-purity bismuth (6N), antimony (6N), and tellurium (6N) were evaporated. A quartz crystal microbalance installed in the main chamber was used to calibrate the fluxes arising from the individual cells. To avoid Te vacancies, a flux ratio of (Bi + Sb):Te = 1:10 was provided with a growth rate of 0.07 QL/min [16]. The substrates were cleaned by means of ultrasonic immersion in acetone and ethanol. Thereafter, they were thermally annealed in a tube furnace at 1040 °C for 90 min. Prior to deposition, the substrates underwent an additional in situ thermal anneal at 550 °C for 60 min to ensure a clean surface. For all the post-annealing procedures, a ramp rate of 5 °C/min was used to reach the annealing temperature and to cool the film back down to the deposition temperature. During the post-annealing, a Te-rich environment was maintained to prevent Te re-evaporation.

The film stoichiometry was determined by in situ X-ray photoelectron spectroscopy (XPS). Using a vacuum suitcase, the films were transferred to an Omicron nanotechnology surface analysis system equipped with a monochromatic aluminum source (K_α_ X-ray source XM1000), while maintaining a pressure below 2.0 × 10^−11^ mbar. The film surface morphology was probed by atomic force microscopy (AFM) using a Bruker ICON Dimension Microscope in tapping mode in air with Bruker TESPAV2 cantilevers. Ex situ high-resolution X-ray diffraction (XRD) measurements and reciprocal space maps (RSM) were carried out using a Bruker D8 Discover diffractometer equipped with an Eiger2 R 500K area detector. Asymmetric RSMs along (0120) and (0119) reflections of BST, together with the (018) reflection of Al_2_O_3_, were obtained in coplanar geometry in grazing-exit configuration. Additional texture studies were performed using a Panalytical X’Pert Pro MRD instrument. Transmission electron microscopy (TEM) lamellae were prepared using an FEI Nova600 Nanolab DualBeam FIB. TEM measurements were carried out on a Philips CM300ST.

## 3. Results and Discussion

### 3.1. Characterization of Pristine BST Films

To understand the effect of a post anneal on the structural properties of the BST film, we will start by characterizing the crystal orientation of a non-annealed film and investigate the influence of the substrate step edge density on the growth of BST thin films. In situ XPS was used to verify the stoichiometry of a 10 nm BST film deposited without employing a post-anneal, giving an atomic concentration of Sb in the (Bi_1−x_Sb_x_)_2_Te_3_ films of *x* = 0.59 ± 0.04. Taking into account the resolution of our equipment, we state that our films exhibit an Sb concentration of x = 0.6.

Due to the weak VdW coupling characteristics for topological chalcogenides, as well as the quasi-VdW growth mode [16], the material is highly prone to twin formation. A twinned domain has a unit cell which can be described by a 180° rotation around the [001] direction, i.e., the unit cells that build up the twin domains are each other’s mirror image. Elaborate studies have been performed on methods to suppress one of these twin domains. Examples of this are (i) decreasing the growth rate [17], (ii) using the two-step growth method (although not presented to be an infallible method) [18], and (iii) depositing the film on a rough substrate surface [19] or (iv) on vicinal substrates [20,21]. Kim et al. [22] showed that the structural rearrangement of atoms near a twin boundary leads to a local modification of the electronic structure, suppressing the carrier mobility. Additionally, they performed density functional theory calculations that showed the appearance of a defect level within the bulk band gap, which can introduce additional carriers into the material. Therefore, we emphasize that a suppression of the twinning of the crystal structure is in our favor as well.

**Figure 1:**
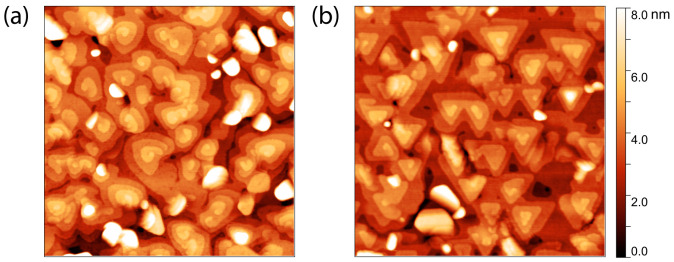
AFM images (1 μm × 1 μm) showing the surface morphology of two 10 nm BST films deposited on Al_2_O_3_ (001), without employing a post anneal. The terrace width of the used Al_2_O_3_ (001) substrates was approximately 270 nm (**a**) versus 60 nm (**b**), which correspond, respectively, to a miscut of 0.07° and 0.33°.

**Figure 2:**
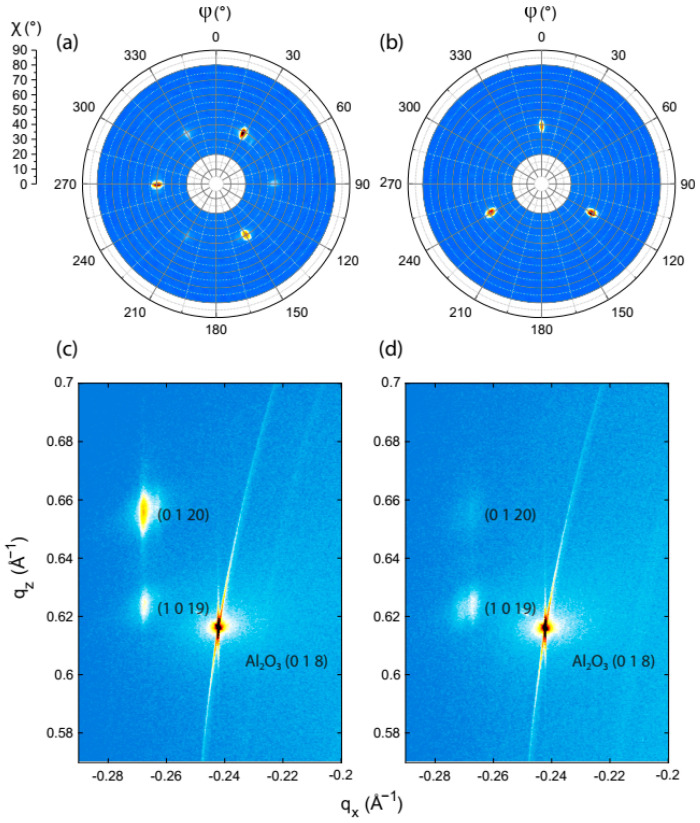
Pole figures taken in symmetric 2θ−ω configuration at 2θ = 38.06°, mapping the BST {1010} for two 10 nm BST films deposited on Al_2_O_3_ substrates with a terrace width of approximately (**a**) 270 nm versus (**b**) 60 nm. ϕ and χ represent the azimuthal and tilt angles, respectively. Their corresponding RSMs are presented in (**c**,**d**), respectively. The inversion observed in the intensities of the BST reflections in the RSM can be attributed to crystal twinning.

Below, we show that the use of substrates with a large vicinal angle allows for the suppression of one of the twin domains. Hence, a large miscut angle is indispensable. We study the formation of twin domains by growing nominal 10 nm BST films on Al_2_O_3_ (001) substrates with two distinct miscuts, i.e., (0.07 ± 0.01°) and (0.33 ± 0.01°), and roughnesses of ~0.6 nm and ~1.9 nm, respectively. Figure 1 presents AFM images of both substrates, revealing the surface morphology of 10 nm BST films on which no post anneal was employed. The film surfaces exhibit archetypical triangular islands with single QL steps, reflecting the rhombohedral crystal structure of the material. The orientation of such triangular islands reflects the presence (absence) of both (one) twin domains on the substrate with a small (large) miscut. This observation is confirmed by the texture analysis performed and presented in the pole figures in Figure 2a,b. These reflect the in-plane orientation of the crystal structure of the 10 nm BST films deposited on Al_2_O_3_. As the two twin domains present each other’s mirror image, their crystal structures are effectively rotated by 60° with respect to each other, see Figure 2a. The presence of only three reflections from the BST {1010} in Figure 2b indicates this BST film does not exhibit twinned crystals, or at least that one twin domain is largely suppressed. Therefore, evidently, this suppression can be realized using a substrate with terraces of approximately 60 nm in width (0.33° miscut).

A plausible cause for this suppression is the higher density of parallel step edges present on the substrate, which can serve as nucleation sites during the growth process. The parallel step edges would align the nucleation sites that favor one (majority) domain orientation. Therefore, when BST islands originate from these nucleation sites and start to coalesce with other islands, the twin domain orientation favored by the substrate orientation will prevail. In the case of segregated nucleation sites, the minority domain has a small window of opportunity, resulting in just a partial suppression of the twinning.

Figure 2c,d presents the RSMs of both films. These RSMs capture the reciprocal space in proximity to the reflection of the Al_2_O_3_ (018) plane in a grazing-exit configuration. By comparing the relative intensities of the BST (0120) and (1019) reflections, we find that the intensities of the peaks invert. The RSM in Figure 2c is in line with previously reported results acquired from Bi_2_Te_3_ deposited on Al_2_O_3_ (001) [23,24]. As mentioned before, one of the main differences between the two films is the crystal twinning. Therefore, we state that the origin of the inversion can be found by looking at the relative intensities and the relative in-plane orientation of the reflections.

The Al_2_O_3_ (018) plane and the BST (0120) and (1019) planes are all three-fold symmetric. To explain the origin of the inversion, we first focus on the relative intensity of BST reflections in our RSMs. Generally, the (0120) reflection is about 26.5 times more intense than the (1019) reflection [25]. Therefore, naively, one would expect the (0120) reflection to exhibit a higher intensity diffraction spot than the (1019) reflection. However, the in-plane orientation of the Al_2_O_3_ (018) reflection matches the one from BST (1019) and has a relative in-plane rotation of 60° with respect to the BST (0120) plane. Therefore, only in case that both twin domains are present does the highly intense (0120) reflection appear at the same azimuthal angle as the Al_2_O_3_ (018) reflection, used to align the BST film for the RSM measurements. When one of the twin domains is highly suppressed, the (0120) reflection will also be highly suppressed in the RSM. Only then does the generally less intense BST (1019) reflection become more prominent in the RSM. In previous work [16], we obtained similar results from Al_2_O_3_ (001), but also from InP (111)A and SrTiO_3_ (111) substrates.

Combining the results acquired using AFM with the results from both the texture study and those of the RSMs, we conclude that increasing the number of substrate terrace edges increases the number of nucleation sites, resulting in a more pronounced suppression of one twin domain. As a consequence, this study is performed using Al_2_O_3_ substrates with a similar miscut of approximately 0.33°.

### 3.2. Effect of Post-Annealing Temperature

The presented results of the single domain 10 nm BST thin film are promising, even without applying any additional improvement procedure. However, the morphology reveals large randomly inclined crystals present in the film that are not oriented in the characteristic [001] direction, represented by the undefined white spots on the archetypical triangular islands of BST [001] in the AFM scans in Figure 1b. The presence of these crystals has been reported before in dissimilar substrates [11,26,27,28,29]. Additionally, the surface presents spiral triangular mounds, which are associated with screw dislocations. Similar results have been reported on other materials that exhibit the same rhombohedral crystal structure as BST, i.e., Bi_2_Se_3_ deposited on a variety of substrates such as GaAs (111)B [30], Si (111) [21], SrTiO_3_ (111) [31], and epitaxial graphene/SiC (001) [32]. Li et al. [21] found that these screw dislocations mainly occur near the twin boundaries and therefore were able to suppress these defects by using vicinal substrates. This is in stark contrast with our results on Al_2_O_3_ substrates, where an increase in the vicinal angle almost completely suppresses one of the twin domains, but spiral triangular mounds are still present. By using a post-annealing procedure, we aim to produce films with an ultra-smooth surface in which these randomly inclined crystals and screw dislocations are no longer present.

Various methods have been proposed and studied to improve the surface morphology and crystalline quality of BST compounds. An example of this is the two-step growth process used by Harrison, S. E. et al. [24], which was employed to minimize the occurrence of the observed randomly inclined crystals. Their process involved growing a 20 nm nucleation layer. This layer is annealed in a Te-rich environment while ramping the substrate temperature up to the temperature used to deposit Bi_2_Te_3_. At this temperature, they continued depositing Bi_2_Te_3_ until they reached their desired film thickness. Li et al. [21] also found the surface smoothness and crystallinity achieved with single-step growth to be inferior compared to that achieved with two steps. Additionally, various studies have shown that increasing the substrate temperature during deposition can increase the lateral dimensions of the triangular mounds for this material. However, in practice, the substrate temperature window is quite narrow. Too low a temperature leads to a reduction in the adatom diffusion, resulting in a polycrystalline or amorphous film, and too high a temperature leads to the formation of 3D islands [33]. Additionally, decreasing the deposition rate increases the time available for surface diffusion, but will in turn favor Te desorption from the surface due to its relatively low sticking coefficient [34]. Therefore, to achieve an ultra-smooth surface morphology, post-annealing is indispensable. Liu et al. [11] showed that they could significantly improve the surface morphology of 30 nm films by annealing the BST film at 307 °C for 4 h, but that employing an annealing temperature of 347 °C led to, what they interpreted as, re-evaporation of the film material.

**Figure 3:**
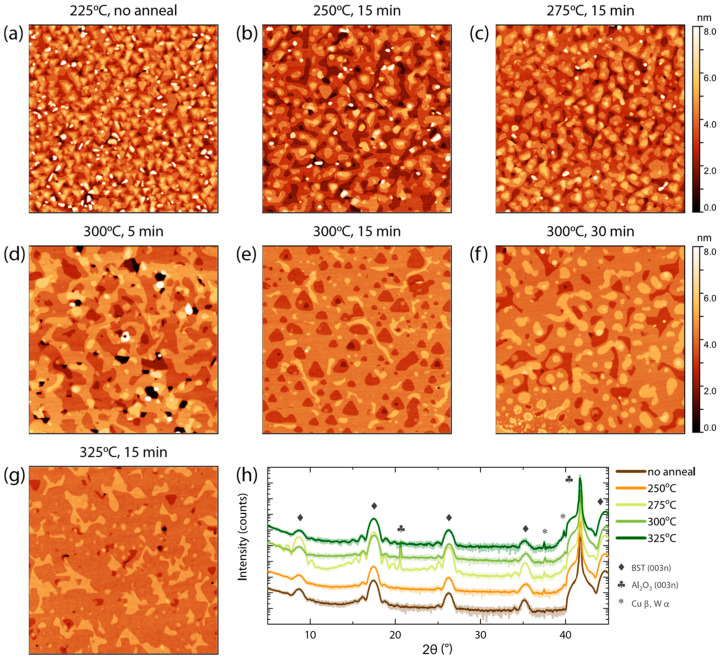
AFM images (3 μm × 3 μm) of 10 nm BST on Al_2_O_3_ (001). (**a**) The morphology for the reference film, without a post anneal. (**b**–**g**) 10 nm BST films on which a post anneal was employed to improve the surface morphology. Employing a post anneal at 300 °C or higher results in a significant improvement in the surface roughness, revealing a homogeneous height distribution. The surface morphology of the annealed film at 15 min and 300 °C still reflects the rhombohedral crystal structure. (**h**) 2θ−ω diffractogram for the film without post anneal and the films annealed for 15 min at different temperatures. Peaks associated with the (003n) BST phase are clearly observed.

For our study, to improve the structural quality and surface morphology of ultra-thin BST films, we employed annealing temperatures of 250 °C, 275 °C, 300 °C, and 325 °C, see Figure 3. All films were deposited at 225 °C. During the post anneal and the cool down from the post-annealing temperature to the deposition temperature, we maintained a Te-rich atmosphere to prevent the out-diffusion of Te [35]. To gain insight into the influence of the annealing temperature on the deposited films, we also deposited BST films at these elevated temperatures. At a growth temperature of 275 °C, we were still able to grow a crystalline BST film. However, raising the growth temperature to 300 °C resulted in no material being adsorbed on the substrate. From the AFM results presented in Figure 3, we conclude that the films still contain buckled crystals when annealing temperatures below this limit, i.e., 250 °C and 275 °C, were empolyed.

To quantify the improvement in surface morphology, we defined the following parameters: (i) the fractional area of randomly inclined crystals, *f*, (ii) the average height of these crystals, *h_c_*, and (iii) the overall RSM roughness, *σ*. For all films, three AFM scans on different surface areas were collected. Setting a threshold height (4 nm higher than the median height of the film), *hc* was determined as the average height of features above this threshold height. The ratio between this area and the total area of the scan is *f*. Comparing the values of parameters found for the films post annealed at 250 °C and 275 °C to the initial film shows a significant reduction in both *f* and *σ* (see Table 1). Figure 3h presents the XRD 2θ−ω diffractogram of the film deposited without a post anneal and the films annealed for 15 min at 250 °C, 275 °C, 300 °C, and 325 °C. For all films, the XRD measurements solely show the reflections corresponding to the Al_2_O_3_ (001) substrate planes and the (003n) planes of BST, confirming the film’s rhombohedral crystal structure. An improvement in the crystal quality is observed upon performing a post anneal at 275 °C. Even so, the surface morphology of this film, as revealed in Figure 3c, still exhibits many QL steps. To be able to perform QSH measurements, we aim to acquire a more homogeneous height distribution. To achieve this, the film needs to be restructured without re-evaporating the material. Therefore, we investigated post-annealing procedures at 300 °C for a variety of annealing times. The AFM results of the different annealing temperatures and times are presented in Figure 3d–f. It is clear from Figure 3e that the material gains enough energy to restructure itself within 15 min. Using XRR, we confirmed that no material has re-evaporated, while the surface roughness is improved. This improvement is verified by the values found for *f*, *h_c_*, and *σ* presented in Table 1.

A comparison of the XRD results for the annealing procedures at 275 °C and 300 °C reveals a reduction in the crystal quality upon increasing the annealing temperature, as the Laue fringes become less pronounced. However, by increasing the annealing temperature to 325 °C, the Laue fringes reappear. This might be caused by an anneal-induced stoichiometry gradient that suppresses the visibility of the Laue fringes on films post annealed at higher temperatures.

**Figure 4:**
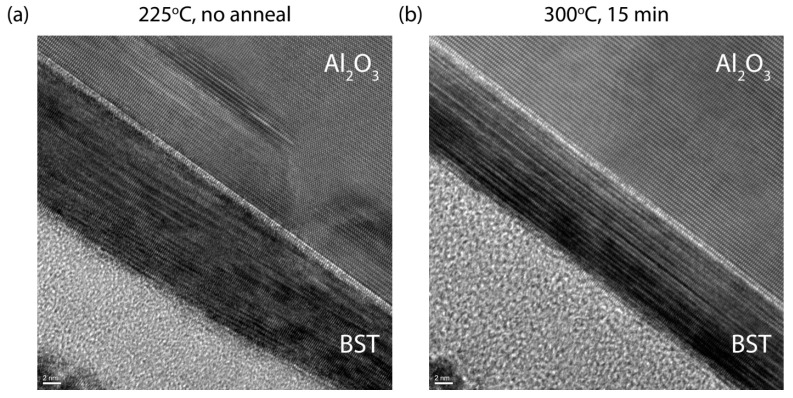
Post-anneal-induced structural improvement in the films visualized by TEM analysis. TEM images presenting a cross-sectional view along the [2-1-10] direction of a 10 nm BST film deposited at 225 °C (**a**) and a 10 nm BST film deposited at the same temperature but post annealed at 300 °C for 15 min (**b**).

To visualize the structural improvement achieved by performing a post anneal, we performed a TEM analysis. Figure 4 compares the TEM results from a 10 nm BST film deposited at 225 °C (a) with a film deposited at the same temperature, but post annealed at 300 °C for 15 min (b). The lamellae were cut in the [2-1-10] direction of the BST film, in which the atomic layers within the QL are stacked in the [001] direction. The annealed film shows a clear QL stacking and a lack of stacking faults, whereas the regular film shows that various QLs are merging.

In situ XPS was performed to verify the material stoichiometry after annealing the films at 275 °C, 300 °C, and 325 °C. We note that we are not drawing any conclusions from the exact stoichiometry, but that these experiments are only performed to verify whether the anneal induces a difference in the Bi:Sb ratio. We observed a variation in the Sb concentration that was slightly larger than individual measurements on BST films with the same stoichiometry. We found that our (Bi_1−x_Sbx)_2_Te_3_ films annealed at 275 °C, 300 °C, and 325 °C for 15 min contained an Sb percentage of *x* = 0.56, 0.54, and 0.54, respectively. No clear deviations were found in the Te percentages. This brings us to the conclusion that even though the effect is very small, the post anneal might have caused some of the Sb to either diffuse or re-evaporate. We speculate that this effect is caused by an imbalance in the formation energies of Bi_2_Te_3_ and Sb_2_Te_3_, where the former forms more easily than the latter [36]. In line with this, we suggest performing a high-resolution TEM study with energy dispersive X-ray spectroscopy to formulate a final conclusion regarding this matter.

### 3.3. Post-Annealing of Ultrathin Films

**Figure 5:**
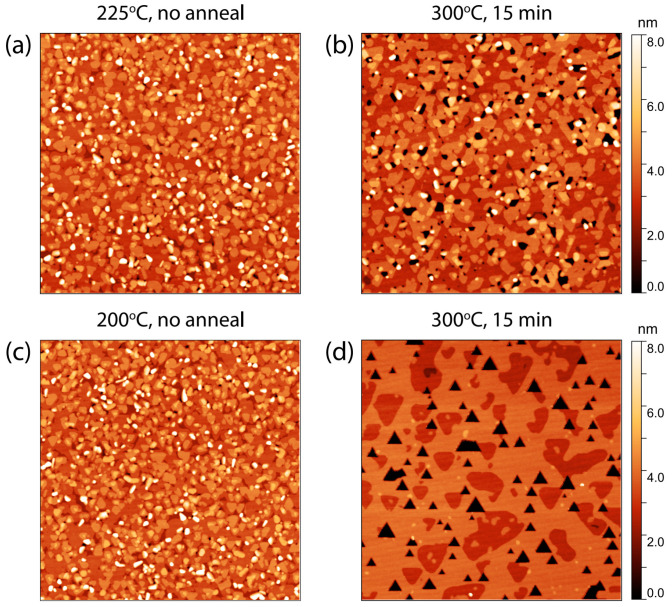
AFM images (3 μm × 3 μm) revealing the surface morphology of 5 nm BST films on Al_2_O_3_ (001) substrates. The AFM images from (**a**,**c**) show the surface morphology for BST grown at 225 °C and 200 °C, respectively. Panels (**b**,**d**) reveal the morphology of BST films deposited at a substrate temperature of 225 °C and 200 °C, respectively, but subsequently post annealed at 300 °C for 15 min.

We have optimized our 10 nm BST films. However, to investigate the QSH state that is predicted to arise in the two-dimensional TI limit, we need to reduce the film thickness to induce a larger hybridization gap [4,5]. Therefore, we performed a similar post anneal study on nominal 5 nm BST films. Figure 5a reveals the surface morphology for a 5 nm film deposited at 225 °C. By applying a post anneal at 300 °C on the 5 nm films, voids are created that reach all the way to the substrate, see Figure 5b. At this stage, the randomly oriented crystals are not yet fully eliminated. To ensure a better nucleation of the BST film in the initial growth process, the same annealing procedure was repeated for a 5 nm film deposited at a lower deposition temperature (200 °C versus 225 °C), to increase the sticking coefficient of the adatoms [24,37,38]. The final result, presented in Figure 5c,d, is striking. The randomly inclined crystals are diminished, the film surface morphology has significantly improved, and the film exhibits a homogeneous thickness. The voids manifest themselves as well-defined triangular holes in the film due to the anisotropic step velocities in the (001) plane of the material [21,32,39], which illustrates the achieved twin domain suppression in these ultra-thin films. These films present the perfect platform for future research on the QSH state of matter in ultra-thin TIs.

**Figure 6:**
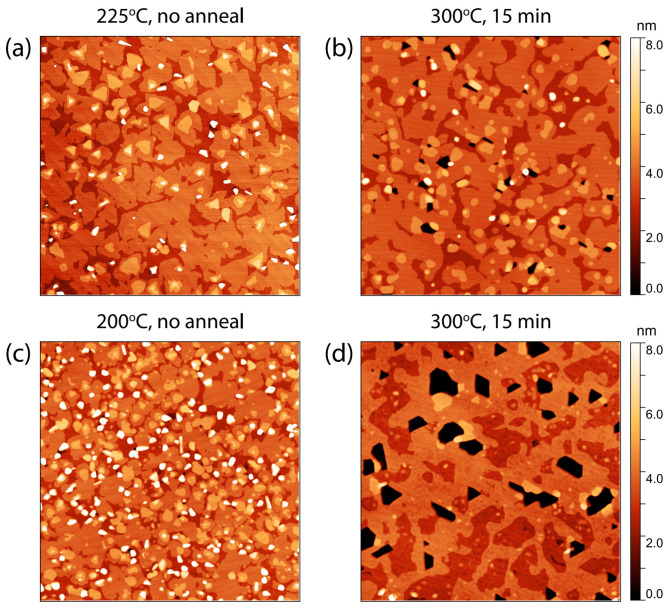
AFM images (3 μm × 3 μm) revealing the surface morphology of 5 nm BST films on SrTiO_3_ (111) substrates. By applying the same optimized procedure on BST films deposited on SrTiO_3_ as previously performed on Al_2_O_3_, we are able to improve the surface morphology significantly. The AFM images from (**a**,**c**) show the surface morphology for BST grown at 225 °C and 200 °C, respectively. Panels (**b**,**d**) reveal the morphology of BST films deposited at a substrate temperature of 225 °C and 200 °C, respectively, but subsequently post annealed at 300 °C for 15 min.

To test the applicability and efficiency of our procedure on other substrates, we repeated the annealing procedures on 5 nm BST films on a SrTiO_3_ (111) substrate. The films were deposited using the same growth conditions. The lattice mismatch between BST and SrTiO_3_ is larger than for BST and Al_2_O_3_, with values of −21.7% and −9.2%, respectively. As a result of this large lattice mismatch, the BST films on SrTiO_3_ generally exhibit a mosaic twist [16], which is reflected in the surface morphology presented in Figure 6a. Additionally, the multi-directional triangular islands reveal that both twin domains are present. Without a post anneal, the BST film shows a large coverage of the substrate surface compared with the film deposited on Al_2_O_3_ substrates.

A comparison between the 5 nm BST films on Al_2_O_3_ and SrTiO_3_ shows that the latter presents a higher level of homogeneity. This is also apparent from the results of the post anneal performed on this film as well, see Figure 6b. However, as the randomly inclined crystals are still present and the film starts to exhibit voids after performing the post anneal, we tried to improve the initial nucleation of the film by decreasing the deposition temperature. Although the surface roughness of the BST film increases upon decreasing the deposition temperature to 200 °C, see Figure 6c, a subsequent post anneal reveals a significant improvement in the surface morphology, see Figure 6d. Similar to the results presented for Al_2_O_3_ in Figure 5d, the void density increases as a result of the post anneal procedure. The shape of the voids reflects the presence of the two twin domains. However, as the twin domains differ by a 60° rotation of the crystal structure, it is self-evident that the post anneal procedure would not impose an improvement in this crystal twinning effect.

## 4. Conclusions

To summarize, we reported the successful growth of high-quality ultrathin 10 and 5 nm BST films on Al_2_O_3_ (001) substrates by MBE. We showed that increasing the miscut angle of Al_2_O_3_ (001) substrates can suppress the occurrence of crystal twinning. The higher density of parallel step edges present on the substrate serves as an aligned nucleation site during the growth process, enhancing one domain orientation. This methodology can be extended to other substrates. Additionally, our post anneal study revealed the enhancement in the BST surface morphology and a reduction in the number of structural defects upon thermal treatment of the films after deposition, reaching RMS roughness values on the order of 0.6 nm. We observed signs that the post anneal induces a small reduction in the amount of Sb in the film. Additional studies on deposit/annealing temperatures are still necessary to achieve an ultra-thin, void-free film. However, as we were able to grow smooth, ultrathin TI films, we are a step closer to future QSH experiments. The range of thicknesses for which Bi_2_Te_3_ and Sb_2_Te_3_ are expected to have the required inverted hybridization gap has been calculated to be a single and three unit cell(s), respectively [5,40]. With the stoichiometry of the present BST, which is closest to Sb_2_Te_3_, we conclude that post-annealed BST on a sapphire substrate is a promising platform for further transport studies, where these films should be structured into submicron structures with multiple contacts.

## Figures and Tables

**Table 1:** Height in nm above the median height of the film. Using the marked area, we determine the average crystal height, *h_c_*.

T_anneal_ (°C)	t_anneal_ (min)	f (%)	h_c_ (nm)	σ (nm)
-	-	2.3 ± 0.3	7.0 ± 1.3	1.15 ± 0.1
250	15	1.3 ± 0.1	6.3 ± 0.2	1.06 ± 0.03
275	15	0.7 ± 0.3	6.5 ± 0.6	1.00 ± 0.04
300	5	0.8 ± 0.6	6.9 ± 1.2	1.43 ± 0.11
300	15	- *	- *	0.63 ± 0.01
300	30	- *	-*	0.68 ± 0.03
325	15	-*	-*	0.49 ± 0.02

* For the 15 and 30 min anneal above 300 °C, *f* was too small to determine both *f* and *hc*.

## Data Availability

All data included in this study are available upon request upon contacting the corresponding author.
